# Bilayer nanographenes: structure, properties, and synthetic challenges

**DOI:** 10.1039/d4cs00804a

**Published:** 2025-10-10

**Authors:** Patricia Izquierdo-García, Juan Lión-Villar, Jesús M. Fernández-García, Nazario Martín

**Affiliations:** a Departamento de QuímicaOrgánica I, Facultad de Ciencias Químicas, Universidad Complutense de Madrid 28040 Madrid Spain nazmar@ucm.es; b IMDEA-Nanociencia 28049 Madrid Spain

## Abstract

Molecular nanographenes (NGs)—graphene analogues at the nanoscale—exhibit atomically defined monodispersity in both size and shape. This synthetic precision enables fine control over their properties. Among the emerging strategies to modulate their electronic and optical properties, vertical π–π stacking between the graphitized layers has recently gained attention as a powerful design tool. In this review, we explore the synthesis, structural features, and functional implications of bilayer and multilayer nanographenes, with a particular focus on the bilayer effect—a through-space electronic communication arising from the interlayer overlap. We discuss how the degree of π–π overlap, rather than solely π-extension, governs key properties such as HOMO–LUMO gap, redox behavior, photoluminescence shifts and quatum yields, and chiroptical responses. Molecular architectures incorporating helicenes, spirocycles, or non-benzenoid motifs enable the deviation from planarity, ususally presented in nanographenes, allowing the precise synthesis of covalently π–π stacked topologies that amplify this effect. Furthermore, this concept also extends to other NGs such as multilayers, supramolecular assemblies, and donor–acceptor complexes, revealing the versatility of the bilayer approach. The first synthetic approaches to access enantiomerically pure bilayer NGs are also disclosed, opening new avenues for their use in advanced technological applications. Overall, the bilayer effect emerges as a novel structural parameter for tuning the properties and function of π-conjugated carbon-based materials, opening new frontiers in molecular chiral optoelectronics, spintronics, and quantum nanoscience.

## Abstract 2

Molecular nanographenes are graphene analogues at the nanoscale. Among the emerging strategies to modulate their optoelectronic properties, vertical π–π stacking between the graphitized layers has recently gained attention as a powerful tool.

## Graphical abstract

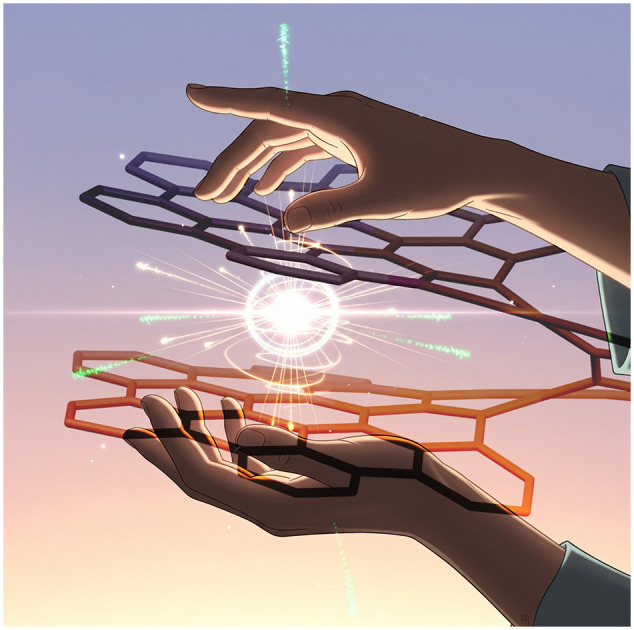

## Introduction

The mechanical exfoliation of graphite-multilayered 3D carbon allotrope-during a “Friday-night experiment” led to the isolation of graphene. The discovery of this honeycomb-patterned monolayer consisting of sp^2^-hybridized carbon atoms by A. Geim and K. Novoselov in 2004,^1,2^ for which they were awarded the Nobel Prize in Physics in 2010, laid the foundation of 2D materials.^3–5^ Interestingly, materials based on fused hexagonal networks of sp^2^ carbon atoms can exhibit remarkably different mechanical and electronic properties depending solely of the number of layers.^6,7^ While bulk graphite exhibits moderate electrical conductivity, especially along the basal planes, single-layer graphene displays remarkably higher charge carrier mobility and a semimetallic character due to massless Dirac fermions.^8^

In 2010, Eva Y. Andrei and collaborators predicted that two stacked graphene layers with a slight twist between them, could create sharp peaks—known as van Hove singularities—in the electronic density of states (DOS).^9^ These peaks amplify the effects of electron–electron interactions and were expected to trigger the emergence of unusual phases of matter, such as superconductivity or correlated insulating behaviour, especially when the system is tuned close to these energy levels. A few years later, in 2018, P. Jarillo-Herrero and his team reported the observation of unconventional superconductivity in graphene bilayers at a ‘magic angle’ of 1.1° and 1.7 K,^10^ effectively establishing the field of twistronics (Moiré patterns).^11^ Once again, the same atomic structure exhibited dramatically different properties depending on both the number of stacked layers and respective orientations.

This remarkable sensitivity of graphene's properties to stacking and orientation invites reflection on another simpler and iconic carbon-based structure: benzene. Discovered by M. Faraday in 1825,^12^ benzene marked the beginning of aromatic chemistry and introduced the concept of delocalized π-electron systems—foundational to modern organic and materials chemistry.^13^ While benzene represents the molecular limit of a conjugated sp^2^-carbon aromatic system, graphene can be viewed as its two-dimensional, periodic counterpart.

**Fig. 1:**
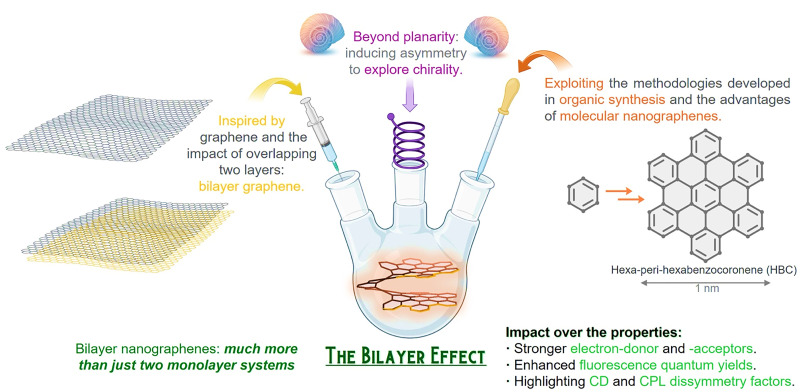
Bilayer nanographenes and the bilayer effect; inspired by graphene, accessible through advanced organic synthetic methodologies that allow going one step beyond atomic precision, controlling the inherent chirality in these sophisticated structures.

Thus, inspired by graphene and employing the tools of organic chemistry involving aromatic systems, nanoscale equivalents known as molecular nanographenes have emerged (Fig. 1).^14^

### Molecular nanographenes: synthesis, properties and chirality

Nanographenes (NGs) are typically defined as small graphene flakes with diameters ranging from 1 nm to 100 nm.^15^ The quantum confinement of electrons and edge effects endow these materials with semiconducting character and lead to the emergence of optoelectronic properties.^16^ Traditionally, nanographenes have been prepared *via* top-down approaches starting from bulk materials such as graphite, graphene, or graphene oxide.^17^ In these methods, physical or chemical cutting processes produce nanographene fragments or graphene quantum dots (GQDs); however, control over their size, shape and functionalization remains limited, often resulting in polydisperse mixtures and poorly defined edge structures without well stablished properties.

Alternatively, the versatility of organic chemistry has enabled the design, and atomically controlled synthesis, of sophisticated molecular nanographenes. Thus, bottom-up organic synthetic strategies enable control over their size, shape, edge, electronic structure.^18–20^ The rational structure–property relationship allows for the systematic tuning of their optoelectronic, magnetic, and redox properties,^21^ making molecular nanographenes ideal candidates for applications in organic electronics, spintronics, and quantum materials.

The benchtop preparation of molecular NGs relies on the strategic assembly of small aromatic precursors through well-defined organic reactions that enable the formation of extended π-conjugated systems. Among the most widely employed transformations, aryl–aryl cross-coupling reactions,^22^ [4+2] Diels–Alder cycloadditions,^23^ and cyclotrimerizations of arylalkynes,^24^ which offer robust and modular routes for constructing large oligoaryl frameworks, are the most successful. Once the molecular backbone is in place, oxidative cyclodehydrogenation—most commonly *via* the Scholl reaction—serves as a key step to induce full planarization (graphitization) of the structure by forming multiple new C–C bonds between adjacent aromatic units.^25–30^

Using this approach, the considered smallest molecular NG (∼1 nm^2^) obtained by organic synthesis is hexa-*peri*-hexabenzocoronene (HBC), firstly described independently by Clar^31^ and Halleux^32^ in 1958—although the term nanographene was not yet coined at that time.

Just like graphite's multilayer structure, planar nanographenes tend to aggregate *via* π–π intermolecular van der Waals (vdW) interactions. Taking advantage of this behaviour, Müllen and collaborators reported, in 1999, the first supramolecular aggregates of HBC derivatives, forming ordered columnar structures.^33^ This finding led to a significant interest in the search for ordered HBC-based liquid crystals in early 2000s, driven by their relevance in organic electronics.^34^

However, it has been in recent years that vdW or covalently linked, discrete, persistent bilayer and multilayer nanographenes have successfully been prepared in a controlled synthetic manner—that is, with a defined number of overlapping layers stacked *via* π–π interactions.

Discrete molecular bilayers held together by vdW interactions offer a minimalist platform to explore interlayer effects. Through careful molecular design—such as edge functionalization or steric modulation—it is possible to direct the assembly toward well-defined persistent bilayer structures, while avoiding uncontrolled multilayer aggregation. Yet, to move beyond non-covalent assemblies and construct covalently linked bilayer or multilayer nanographenes, one must overcome the intrinsic planarity imposed by the hexagonal graphene lattice—essentially ‘rising from flatland’.^35^ Breaking this strict two-dimensionality enables vertical stacking of discrete layers. However, this structural deviation from planarity is not merely a geometric requirement, since it has also profound implications at the topological level. In particular, non-planar distortions often lead to the emergence of chirality in the resulting molecular NGs, giving rise to enantiomeric forms with distinct optical activity.^36^ Consequently, such systems may exhibit chiroptical properties—including circular dichroism (CD) and circularly polarized fluorescence (CPL)—opening new avenues for their application in areas such as enantioselective sensing, chiral optoelectronics,^37^ and chiral-induced spin-selectivity (CISS).^38^

In this regard, starting from hexagonal symmetry compounds, it is possible to access non-planar NGs endowed with curved architectures.^39–41^ The presence of non-hexagonal rings—such as pentagons, heptagons, or octagons—into the π-conjugated backbone disrupts the flat geometry of the honeycomb lattice, giving rise to bowl- or saddle-shaped surfaces. These 3D NGs often exhibit interesting electronic and optical properties resulting from the interplay between curvature and conjugation.^42^ Notably, some of these structures are inherently chiral, exhibiting chiroptical properties determined by circular dichroism (CD) and circularly polarized luminescence (CPL).^43^ However, the out-of-plane distortion introduced by these non-hexagonal motifs is generally not enough to promote the formation of discrete bilayer or multilayer molecular NGs. In this regard, formation of stacked multi-level architectures, requires the presence of strongly distorted fragments—such as helicenes or spirocycles—that enforce a larger spatial separation and orientational control between the layers.^44^

In this review, we explore the synthetic strategies behind precisely designed bilayer and multilayered molecular nanographenes, including the first enantioselective accomplishments, with particular emphasis on how the number of layers, their degree of π-overlap, and relative orientation, influence their properties. Thus, we define the ‘bilayer effect’ as the phenomenon arising from the interaction between π-layers, which plays a key role in the emergence and modulation of the resulting properties.

## Helicene-based layered nanographenes

Among the different structural motifs employed to construct vertically organized layers of nanographene architectures, helicenes stand out for their helical topology and inherent chirality.^45^ Their twisted, non-planar structure not only enables controlled spatial separation between π-systems but also introduces directional stacking preferences that are advantageous for designing bilayer and multilayer systems. In this section, we explore how helicene units have been strategically integrated into molecular NG frameworks to access a diverse range of layered materials, highlighting structural features and the impact of helicity on the resulting optoelectronic and chiroptical properties.

### Helical bilayer nanographenes

**Fig. 2:**
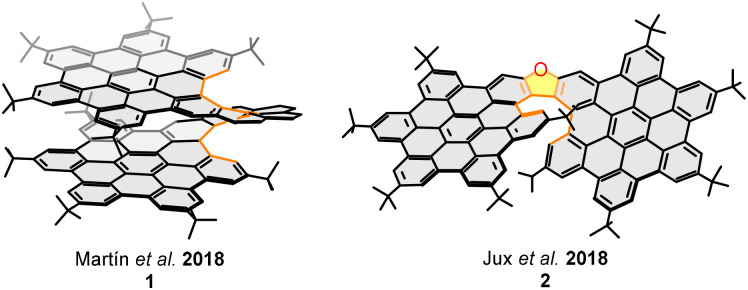
Helical bilayer nanographene 1 and π-extended oxa[7]helicene 2.

Our research group, in collaboration with Crassous’ group, were pioneered in describing the first helical bilayer nanographene in 2018. Molecular bilayer nanographene 1 (Fig. 2) featured a [10]helicene joined to two HBC units, resulting in a totally conjugated [10]helical bilayer system.^46^ The synthesis was carried out in a multistep synthetic procedure from dialkynyl[6]helicenes involving sequential Sonogashira, [4+2] Diels–Alder cycloaddition and Scholl reactions. The helical feature's formation is key; it not only twists the structure away from planarity, thereby inducing chirality, but also arranges the layers face-to-face with a 3.6 Å separation. Later that same year, Jux and colleagues reported nanographene 2 (Fig. 2), which integrates an oxa[7]helicene scaffold with two fused HBC layers.^47^ In contrast to our system, the key difference in this molecule lies in the structure of its helical backbone. The overall length of the helix and the presence of five-membered rings control the degree of overlap between the graphitized layers, which in turn affects both the electronic properties and the chiroptical behavior of the nanographene.

The number of turns of a helicene (defined as the number of 360° cycles),^48^ along with the inter-layer overlap at its ends, is directly influenced by the number and topology of the *ortho*-fused rings. When five-membered rings are present, they open the helicene's inner rim, thereby lessening the number of turns. This explains why [10]helical nanographene 1 exhibits a partially overlapped structure, while the helicene's length and shape in oxa[7]helical nanographene 2 prevents the overlap.

**Fig. 3:**
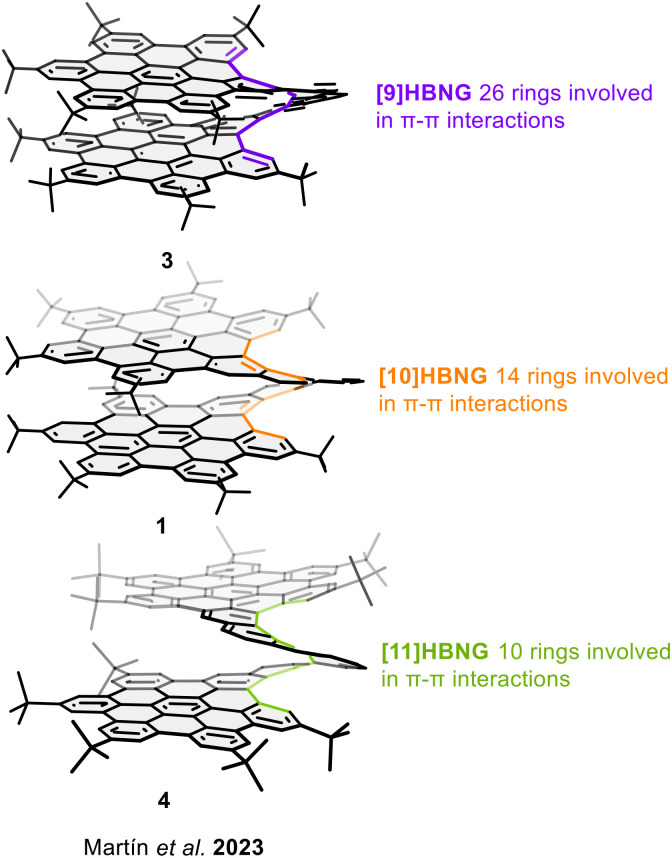
Helical bilayer nanographenes with different helicene lengths: impact of the overlapping degree.

In 2023 our research group, in collaboration with Crassous and Stará's groups, reported a systematic study of the optoelectronic properties of a family of helical bilayer nanographenes (HBNGs, Fig. 3). Three helical bilayer nanographenes with different helicene lengths were synthesized, specifically [9]HBNG (3), [10]HBNG (1), and [11]HBNG (4).^49^ The number of *ortho*-fused rings forming the helicene features determines the extent of the face-to-face organization of the graphitized layers at the distances of the π–π stacking. This influences the overlapping and the resulting optoelectronic properties. The overlapping degree between the graphitized layers was evaluated by single crystal X-ray diffraction (SCXRD) structures. The number of rings involved in the intramolecular π–π stacking decreases with increasing the helicene lengths; 26 benzene rings (13 rings per layer) are involved in the π–π interactions for [9]HBNG (3) with an interlayer average distance of 3.63 Å, 14 rings for [10]HBNG (1) with an average interlayer distance of 3.54 Å, and 10 rings for [11]HBNG (4) with an average interlayer distance of 3.41 Å.

**Fig. 4:**
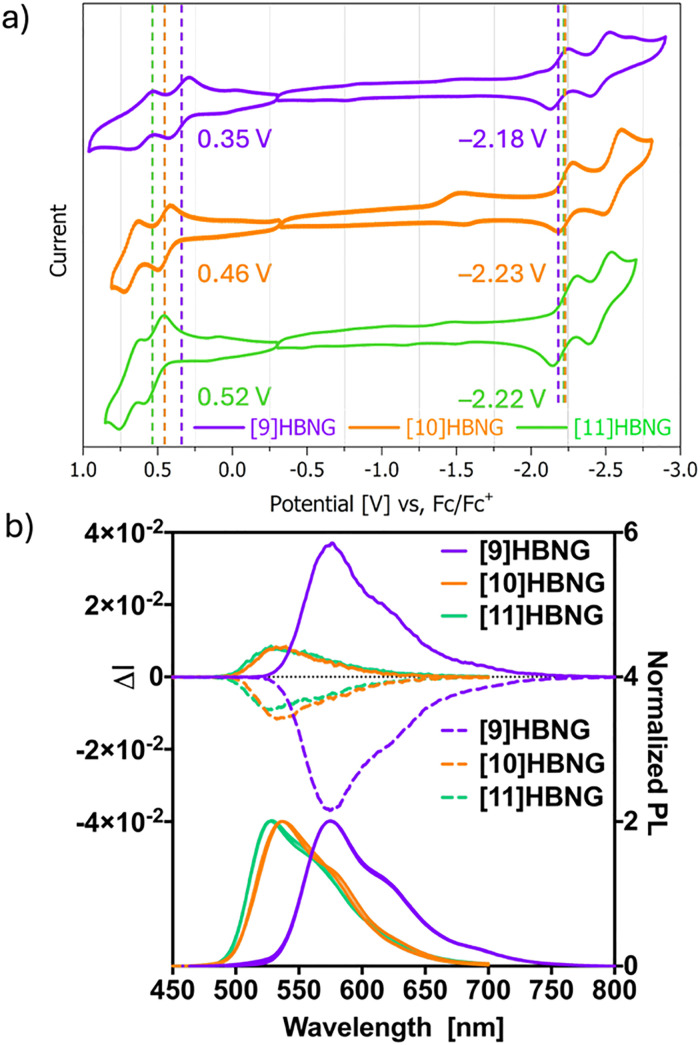
Cyclic voltammograms of [9]HBNG (3), [10]HBNG (1) and [11]HBNG (4) showing the oxidation and reduction behaviour influenced by the overlapping degree. (b) CPL and emission spectra of [9]HBNG (3), [10]HBNG (1) and [11]HBNG (4), showing a red-shifted emission with high *g*_lum_ for the most overlapped nanographene 3.

Despite its lower π-extension, [9]HBNG (3) exhibits the strongest electron donor character. As shown in Fig. 4 the first oxidation potential follows the order [9]HBNG (3) (*E*^1/2^_ox1_ = 0.35 V) < [10]HBNG (1) (*E*^1/2^_ox1_ = 0.46 V) < [11]HBNG (4) (*E*^1/2^_ox1_ = 0.52 V), which is opposite to what would be expected based solely on π-extension. A similar trend is observed for the first reduction potentials: [9]HBNG (3) (*E*^1/2^_red1_ = −2.18 V) < [10]HBNG (1) (*E*^1/2^_red1_ = −2.23 V) ∼ [11]HBNG (4) (*E*^1/2^_red1_ = −2.22 V). These results indicate a narrowing of the HOMO–LUMO gap as the degree of interlayer overlap increases. This behaviour was also observed in the fluorescence emission measurements. The lower π-extended and most overlapped structure, [9]HBNG (3), presents the most red-shifted emission bands, attributed to its enhanced interlayer electronic communication. Furthermore, spectroelectrochemical measurements revealed a mixed-valence band effect for [9]HBNG (3). Thus, the radical cation and cation species are stabilized between the π–π stacked layers.

Regarding the chiroptical properties, this family of HBNGs exhibit exceptional performances, among which [9]HBNG (3) displays one of the highest circularly polarized luminescence (CPL) dissymmetry factors (|*g*_lum_| = 3.6 × 10^−2^) reported for all-carbon polycyclic aromatic hydrocarbons. This remarkable value arises from an optimal alignment between its electric and magnetic transition dipole moments, a consequence of its highly overlapped bilayer configuration, as revealed by theoretical calculations. Thus, the study highlights the pivotal role of helicene length in tuning the photophysical and chiroptical properties of HBNGs.

So far, the most used strategies in molecular nanographenes and other polycyclic aromatic hydrocarbons to modify properties have been: (i) variation of the π-extension, (ii) introduction of non-hexagonal rings and, (iii) doping with heteroatoms. Herein, we aim to introduce and highlight covalently bonded overlapped layers as a new approach to modulate properties depending on the π–π overlapping degree.

The structures considered are those consisting of two graphitized layers arranged at the distance of the π–π stacking. The overlapping degree between these layers is key and can be described by accounting for the number of rings involved in through-space π–π interactions. Greater overlap results in a greater effect on the properties, if the overlap is insufficient, the properties will be governed by other structural features such as π-extension. This π–π interaction has a major impact on the redox properties, the stabilization of oxidized and/or reduced species between the layers enhances the electron-donor and/or acceptor character. The greater the overlap, the greater the effect on the electron-donor and/or acceptor character. The strategies to determine the bilayer effect are mainly: the structural study by SCXRD or DFT calculations, which allow to determine the number of rings involved in the π-interactions and the through-space communication between the layers. If the study does not include a comparison between similar structures with different overlapping degrees where the influence can be systematically observed, it is necessary to carry out spectroelectrochemical measurements (*in situ* registration of UV-vis-NIR spectra upon oxidation or reduction) that allow to determine the mixed valence band character. Furthermore, strong van der Waals interactions between the layers, due to large overlapping degree, can enhance the fluorescence quantum yields; the increase of the molecular rigidity typically suppresses the non-radiative deactivation.

Thus, we could consider the bilayer effect as a new design tool and structural feature, whose effect can have a greater influence over the properties than usual approaches. In addition, chiral bilayer nanographenes based on helicene linkers have shown exceptional performances in chiroptical properties as circular dichroism and circularly polarized luminescence, with high dissymmetry factors. Due to their wide-ranging potential applications,^50^ CPL-active materials^51^ have drawn considerable attention. Organic molecular CPL emitters, present |*g*_lum_| values in the order of 10^−5^ to 10^−2^, in this case, helical bilayer nanographenes present values ranging between 10^−3^ to over 10^−2^.^52^

**Fig. 5:**
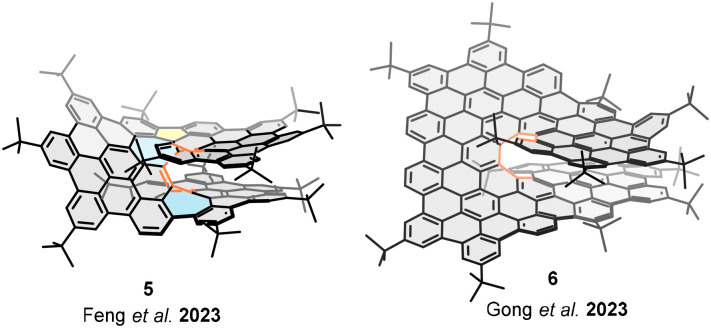
π-extended helical bilayer nanographenes.

The electronic communication between the layers and the highlighting chiroptical properties have brought chiral bilayer nanographenes into the spotlight in recent years. In 2023 Feng and coworkers reported the π-extended non-benzenoid [10]helical bilayer nanographene 5 (Fig. 5).^53^ The structure based on three *pseudo*-HBC fused by five- and seven-membered rings leads to the formation of a [10]helicene. The presence of two seven-membered rings in the helicene framework forces the inner rim to close—in contrast to a [10]helicene formed solely by hexagonal rings—resulting in complete overlap between the layers and a short interlayer distance of 3.24 Å. Oxidation of 5 monitored by *in situ* UV-vis-NIR spectroscopy revealed an intervalence charge transfer band in the NIR region, highlighting the interaction between both layers. These observations were further supported by DFT calculations, which confirmed effective through-space electronic communication between the stacked layers. Moreover, these helical nanographenes display notable chiroptical properties, namely circular dichroism (CD) and circularly polarized luminescence (CPL), quantified by their respective absorption (*g*_abs_) and emission (*g*_lum_) dissymmetry factors. Thus, a luminescence dissymmetry factor |*g*_lum_| of 1.3 × 10^−3^ was determined for enantiomers of nanographene 5.

In 2023, Gong and co-workers reported helical bilayer nanographene 6, a structure featuring a pentadecabenzo[9]helicene core with four integrated HBC units (Fig. 5).^54^ This structure's rigidity, stemming from the helicene π-extension and significant overlap (28 benzene rings involved in the π–π interactions), yields a tight interlayer distance of 2.9 Å. Remarkably, high values of *g*_lum_ = 4.5 × 10^−2^ for the *P* isomer, and *g*_lum_ = −4.22 × 10^−2^ for the *M* isomer were determined for nanographene 6.

### Helical multilayer nanographenes

**Fig. 6:**
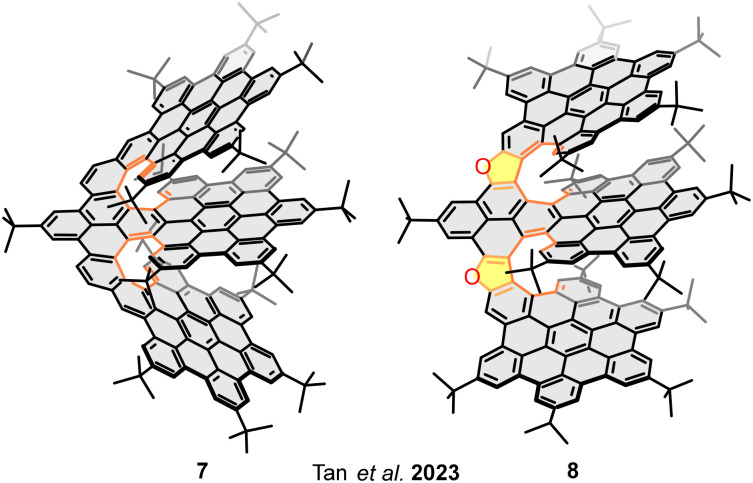
Helical trilayer nanographenes with overlapping degrees depending on the helicenes rings-topology.

In addition to the study of bilayer molecular nanographenes due to their highlighting chiroptical properties, the last years have witnessed a boom in structure/property relationship studies on bilayer and multilayer nanographenes. Hence, in 2023 Tan and co-workers reported the trilayer nanographenes 7 and 8 (Fig. 6), consisting of three HBC moieties connected by [8]helicenes with different topologies.^55^ As mentioned above, the incorporation of five-membered rings in the helicene causes the inner rim to open, directly related to the overlapping decrease. Therefore, although the number of rings forming the helicenes in both structures is the same, [8]helicenes, the five-membered rings in 8 cause a decrease in the overlapping between the layers. The determination of the optoelectronic and electrochemical properties showed that three-layer nanographene with higher interlayer overlapping 7, has a lower first oxidation potential (*E*^1/2^_ox1_ = 0.18 V and *E*^1/2^_ox1_ = 0.25 V, corresponding to 7 and 8, respectively), stronger bound excitons (calculated to be 1.70 eV for 7 and 1.47 eV for 8), and longer photoluminescence lifetimes (10.2 ns for 7 and 7.0 ns for 8), thus demonstrating the relationship between the overlapping degree and the optoelectronic properties. Therefore, the previously defined bilayer effect can be extended to multilayer structures, the number of rings involved in the π–π interactions determine the variation of the redox properties. More overlapped structures are stronger electron donors due to a higher stabilization of the oxidized species between the layers.

**Fig. 7:**
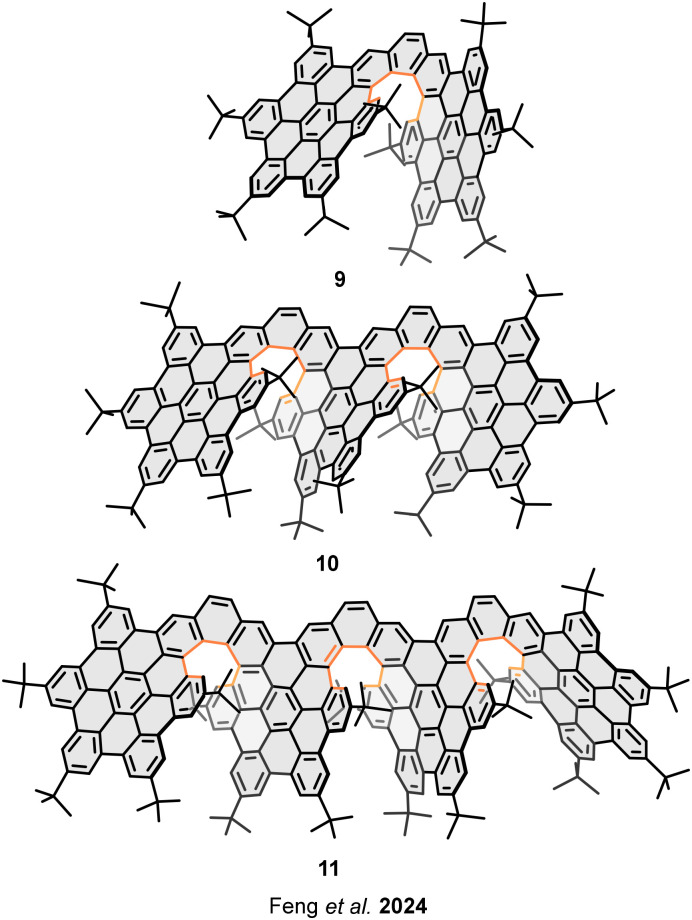
Helical nanographenes with different number of layers.

Further than the comparative study of the properties depending on the overlapping degree, Feng *et al.* have reported three helical nanographenes, in which the overlapping degree between contiguous layers remains constant while the number of layers and the π-extension increase (Fig. 7).^56^ In this case, the multilayered structures based on covalently connected HBC units forming [7]helicenes, exhibit low overlapping degrees between contiguous layers. However, the global π–π intramolecular interactions increases with the number of layers, attributable to the accumulated interlayer interactions. Higher number of layers enhances the rigidity of the structures, which reduces the interlayer spacing. Thus, the distances are 3.78 Å in bilayer 9, 3.26 Å and 3.57 Å in trilayer 10, and 3.56 Å, 3.67 Å and 4.20 Å for the contiguous layers in tetralayer 11 (from left to right in Fig. 7). The incremented π-extension at higher number of layers seems to be the structural reason for the properties’ variation. Tetralayer nanographene 11 exhibits more red-shifted emission bands, preceded by trilayer 10 and bilayer 9. This behaviour is also observed in the bandgap variation; the lowest value corresponds to the tetralayer 11. Furthermore, the electrochemical properties show that the lowest oxidation potential corresponds to the most π-extended structure 11, which exhibits the strongest donor character. Interestingly, this family of nanographenes present highlighting fluorescence quantum yields, 45% for bilayer 9, 74% for trilayer 10, and 91% for tetralayer 11. This trend in the quantum yields may be related to the number of layers and the consequent rigidity enhancement. The chiroptical properties revealed gradually declined values of the luminescence dissymmetry factors (|*g*_lum_|) with the increasing number of layers, 7.9 × 10^−3^ for bilayer 9, 2.6 × 10^−3^ for trilayer 10, and 1.5 × 10^−3^ for tetralayer 11.

**Fig. 8:**
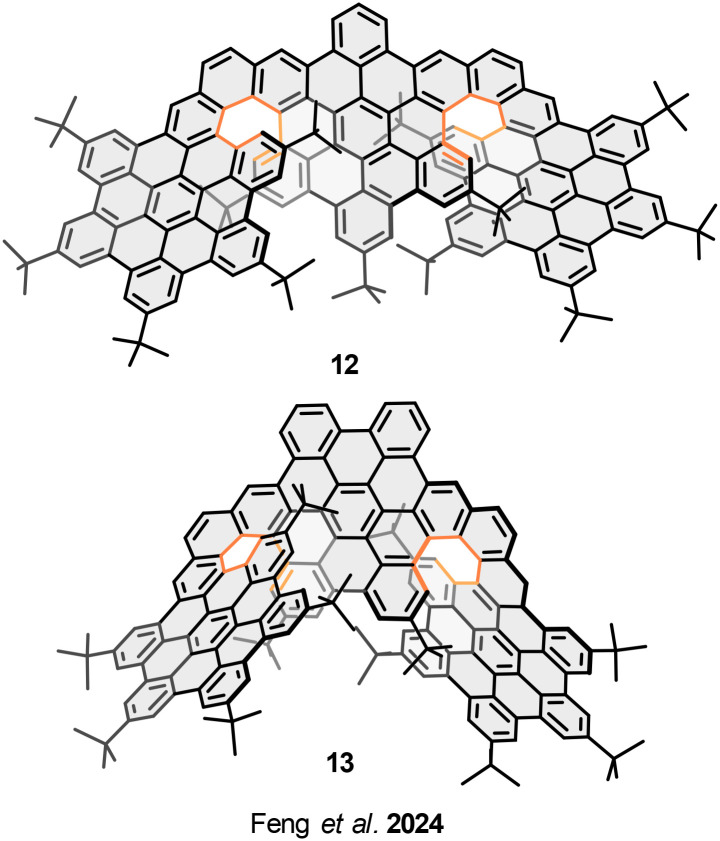
Regioisomeric helical trilayer nanographenes.

Also, Feng and coworkers have described the structure/property relationship between three structural isomers of trilayer nanographenes consisting of three HBC units connected by two [7]helicenes. The modification of the relative fusion positions between the helicenes and the central benzene ring, affords three different geometries, *ortho*-fused nanographene 10 (Fig. 7), *meta*-fused nanographene 12, and *para*-fused nanographene 13 (Fig. 8).^57^ Interestingly, the structural modifications entail the amplification of the chiroptical responses, affording high CD and CPL dissymmetric factors; |*g*_lum_| values, 8.7 × 10^−3^ for *M*-12, and 13.2 × 10^−3^ for *P*-13. A 3.2-fold and 4.8-fold amplification compared to the |*g*_lum_| value of *ortho*-10. While the electrochemical properties show similar electron donor character (*E*^1/2^_ox1_ = 0.36 V), the π-extension remains the same and the π–π interactions between the layers involve the same number of rings; therefore, the overlapping degree between the layers is constant.

These recent results are evidence of the growing interest in bilayer and multilayer nanographenes. Just as deviation from planarity by incorporating topological defects entails the induction of chirality, it is feasible to think that enhancing through-space intramolecular π–π interactions represents a new dimension in the design of nanographenes with advanced properties. A leap out of the designing toolbox in which most structures have so far been encompassed, which only considered modifications in the hexagonal network topology and the π-extension.

**Fig. 9:**
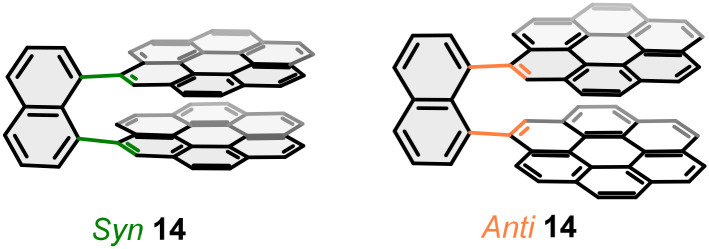
Coronene-based bilayer structure 14 with atropisomerism.

As an alternative to HBNGs, whose chirality is determined by helicene structures, in 2021, Aratani and coworkers described bilayer structure 14 with atropisomerism (Fig. 9).^58^ The structure consists of two coronene units orthogonally connected to 1,8-naphthalene. The restricted rotation of the coronenes due to the overlap of the layers, at around 3 Å distances, gives rise to atropisomerism-type chirality, with an achiral *syn* isomer and a chiral *anti* isomer-with two enantiomers. Absorption measurements showed a slight red shift of the bilayer systems compared to the coronene monolayer. A more significant difference is observed in the emission spectra, where the bands of the bilayer systems show broad bands-which could be correlated with excimer-like emission-with a red shift of between 25 and 50 nm compared to the coronene monolayer. In addition, the enantiomers of the *anti*-isomer showed remarkable |*g*_lum_| values of the order of 1.2 × 10^−3^ and 2.0 × 10^−3^.

### Stereocontrolled synthesis of bilayer nanographenes

As noted in the previous sections, the defining characteristics of the bilayer nanographenes described so far lie in their remarkable chiroptical properties. However, accurate determination of these properties requires the use of enantiomerically pure samples. This requires isolation of the enantiomers by expensive and time-consuming chiral HPLC separation, typically yielding only a few milligrams of material. As a result, this strategy often limits the wider use of these materials in advanced applications.

**Fig. 10:**
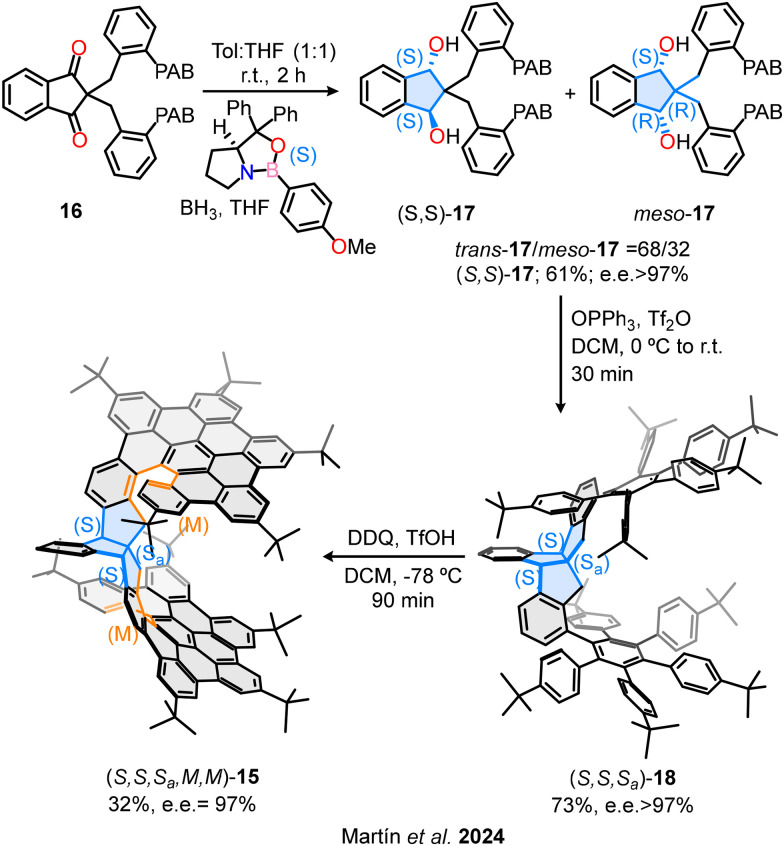
First enantioselective synthesis of a chiral bilayer nanographene.

With the main motivation of avoiding the separation of the enantiomers, our research group has recently described the first enantioselective synthesis of an inherently chiral bilayer molecular nanographene 15 composed exclusively of carbon atoms (Fig. 10).^59^ The route overcomes significant challenges inherent in controlling chirality in π-conjugated, heteroatom-free systems. The synthetic approach consists of a three-step stereocontrolled strategy to introduce central, axial and helical chirality into a rigid polycyclic aromatic framework. Key steps include: (1) enantioselective Corey–Bakshi–Shibata reduction of a prochiral indandione 16 to generate *trans*-diol (*S*,*S*)-17 with high enantiomeric excess; (2) an enantiospecific benzylic substitution *via* a Friedel–Crafts-type cyclization using Hendrickson's reagent, yielding the triindane structure 18 while preserving chiral information; and (3) an enantiospecific Scholl reaction that establishes the final helicoidal chirality and induces stereocontrolled graphitization. The resulting nanographene 15 exhibits remarkable stereochemical stability and excellent enantiomeric excess (e.e. > 97%), as well as unique chiroptical properties, with a CPL dissymmetry factor of |*g*_lum_| = 1.9 × 10^−3^. Therefore, this enantioselective route represents a significant advance in the synthesis of optically active carbon-based nanostructures without relying on pre-functionalized chiral substrates or HPLC resolution. Furthermore, it demonstrates the successful integration of multiple stereogenic elements within a π-extended system, providing a rational methodology for the asymmetric synthesis of complex chiral nanographenes.

**Fig. 11:**
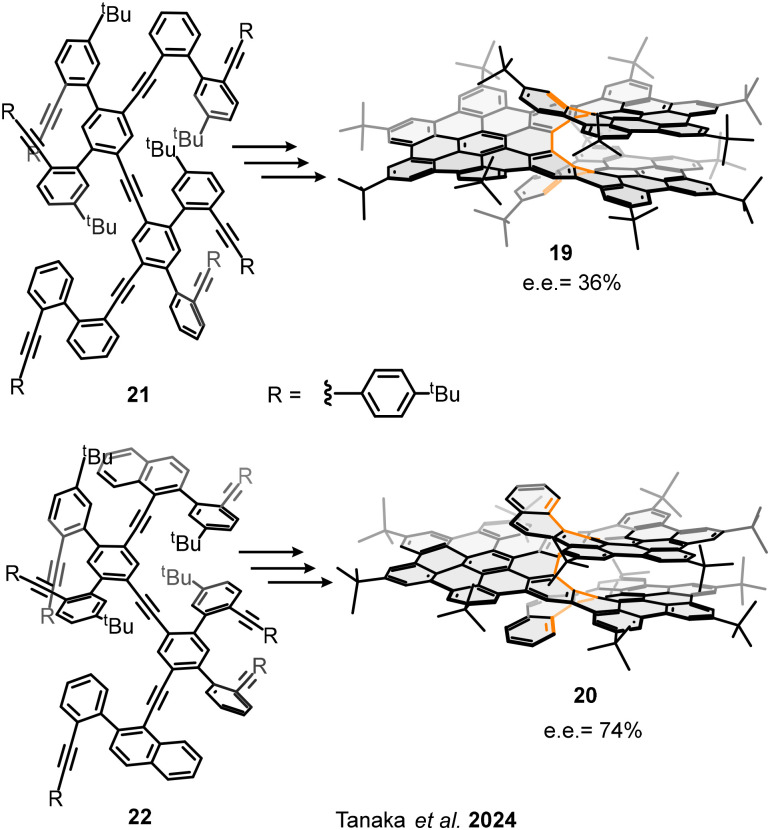
Enantioselective syntheses of chiral bilayer nanographene.

The enantioselective synthesis of molecular nanographenes is a defying endeavour from design to synthesis. To the best of our knowledge, only one more group has described this approach. In 2024, Tanaka and coworkers described the smart synthesis of [11] and [13]helicene based bilayer nanographenes 19 and 20, respectively.^60^ The structures consist of one HBC connecting two truncated HBC units, resulting in large helicene-based bilayer nanographenes, (Fig. 11). The synthetic approach starts with sequential Sonogashira coupling reactions to achieve the precisely designed nonayne precursors 21 and 22. Then, the first key reaction is an enantioselective [2+2+2] cycloaddition in presence of Ni(cod)_2_ combined with (*R*)-1-(2-diphenylphosphino-1-naphthyl)isoquinoline ((*R*)-Quinap), which transfer the chiral information. In this step, the enantiomeric ratio is fixed by the formation of the helical backbone. Then, consecutive Scholl reactions are performed in presence of FeCl_3_ and/or DDQ/TfOH or MsOH to controllably afford the completely graphitized [11]helical bilayer nanographene 19 and [13]helical bilayer nanographene 20 with moderate enantiomeric excesses, 36% and 74%, respectively. The interlayer distances between the overlapping layers are around 3.23 Å, comparable to totally overlapped bilayers connected by π-extended helicenes, as structure 5. The rigidity of nanographenes 19 and 20, provided by the π-extended helicenes and the broad interlayer van der Waals interactions between the overlapped layers (evaluated by X-ray diffraction structures and NCIPLOTs DFT calculations), has a great impact over the photophysical properties. Non-radiative deactivation is suppressed, enhancing the fluorescence quantum yield (*Φ* = 23–31%), thus, reflecting the influence of the strong intramolecular π–π interactions over the properties. The chiroptical properties of nanographenes 19 and 20 were evaluated by electronic circular dichroism (ECD) and CPL spectroscopies. Both structures present high absorption and emission dissymmetry factors, |*g*_abs_| = 2.5–4.0 × 10^−2^ and |*g*_lum_| = 3.4–4.0 × 10^−2^, and high CPL brightness *B*_CPL_ = 490–513 M^−1^ cm^−1^.

**Fig. 12:**
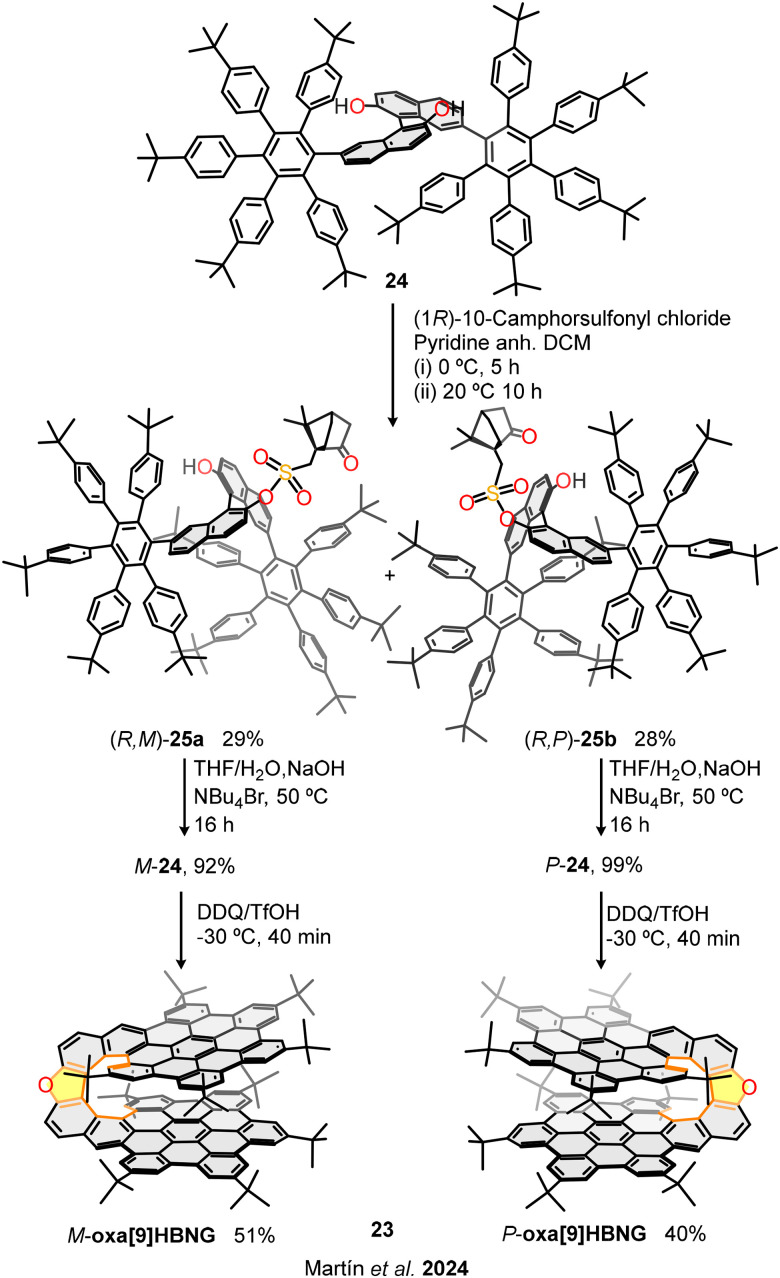
Straightforward stereocontrolled synthesis of helical bilayer nanographenes oxa[9]HBNG based on the chemical resolution strategy.

As described so far, the enantioselective synthesis of nanographenes can be extremely challenging and synthetically demanding. Therefore, our research group has recently described an alternative and straightforward approach for the stereocontrolled synthesis of the helical bilayer nanographenes *M*- and *P*-oxa[9]HBNG (23, Fig. 12).^61^

This study introduces an efficient and scalable synthetic methodology for the preparation of enantiomerically pure helical bilayer nanographenes (HBNGs), overcoming traditional dependence on HPLC for chiral resolution. The presented approach is notable for its simplicity, accessibility, and potential for scale-up, starting from commercially available 7-bromo-2-naphthol. Key to the process is the incorporation of a BINOL core, which provides configurational stability and enables straightforward diastereomeric resolution of the polyarene precursor 24*via* esterification with enantiopure camphorsulfonyl chloride. Diastereomers (*R*,*M*)-25a and (*R*,*P*)-25b are easily separated by standard silica gel chromatography, avoiding complex purification techniques (chemical resolution). Subsequent mild hydrolysis and an enantiospecific Scholl cyclodehydrogenation yield enantiomerically pure *M*- and *P*-oxa[9]HBNGs (23) with >99% enantiomeric excess. This enantiospecific graphitization step results in the axial-to-helical chirality transfer, forming a rigid bilayer structure with significant π–π overlap.

The overlapping degree was determined from single-crystal X-ray-diffraction structures, being 20 rings involved in the π–π interactions, with average distances of 3.6 Å. This large overlapping is slightly lower than that of the all-carbon structure [9]HBNG (3) (26 rings involved in the π–π interactions), since the five-membered ring embedded in the helicene moiety opens the inner rim decreasing the overlapping. As expected, nanographene oxa[9]HBNG (23) exhibits strong electron-donor character showing a first oxidation potential of *E*^1/2^_ox1_ = 0.37 V, close to that of totally overlapped structure [9]HBNG (3) at *E*^1/2^_ox1_ = 0.35 V. Regarding the chiroptical properties, oxa[9]HBNG (23) presents high dissymmetry factors in circular dichroism and circularly polarized luminescence, |*g*_lum_| = 2.5 × 10^−3^. Therefore, this scalable and accessible methodology provides a valuable strategy for the synthesis of complex chiral nanographenes, opening new opportunities in optoelectronics, spintronics, and chiral sensing applications where their unique bilayer architecture and chiral properties can be exploited.

### Functionalized donor–acceptor bilayer nanographenes

The interaction between electron acceptor (EA) and electron donating (ED) units in organic molecular systems have a profound effect on their final properties. From a fundamental viewpoint, EA and ED units communicate through different electronic phenomena which include charge transfer (CT), electron transfer (ET) and energy transfer (EnT) processes.^62,63^

Electron transfer processes yield charge-separated (CS) states that relax through non-emissive pathways but are very relevant for certain applications like organic photovoltaics or artificial photosynthesis.^64,65^ On the contrary, EnT and CT may provide access to advanced optical properties such as thermally activated delayed fluorescence (TADF) or room-temperature phosphorescence (RTP) thanks to the promotion of intersystem crossing (ISC) and reverse intersystem crossing (RISC) processes through narrowing the singlet–triplet energy gaps.^66–68^ These optical features are highly demanded for the development of third-generation OLEDs.^69^

A wide variety of D–A systems which incorporate PAHs or nanographene fragments have been developed in the last decades, including supramolecular^70,71^ and covalent complexes.^72–78^ However, only a few of these systems present a bilayer or multilayer 3D disposition; as it is developed throughout this section, the bilayer arrangement is decisive for the optoelectronic features derived from D–A molecules.

In the past years, Würthner and co-workers have been developing strategies to prepare bilayer and multilayer nanographenes stabilized by van der Waals interactions. The synergetic combination of π–π stacking and interlocking peripheral bulky groups provide access to stable structures able to crystallize and be examined by SCXRD.

**Fig. 13:**
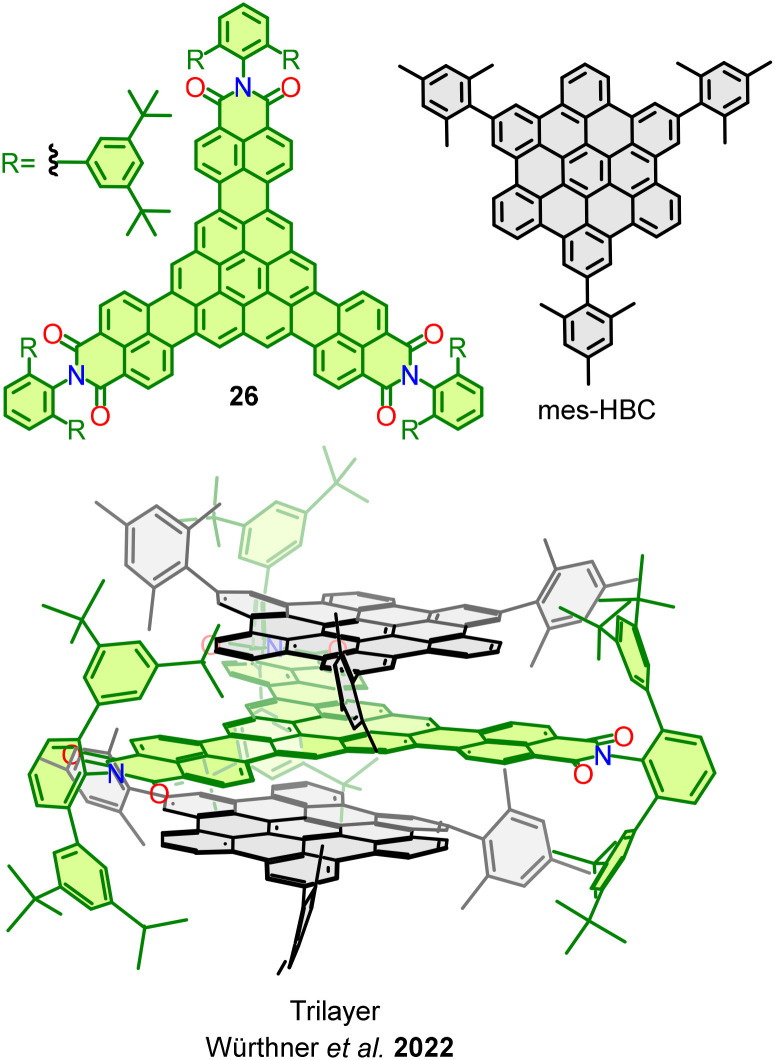
Trilayer structure formed by trisimide nanographene 26 and two mes-HBC.

In 2022, trisimide nanographene 26 was reported.^79^ Although homoaggregates of this NG were observed, it preferentially stacks together with HBC units that give rise to a trilayer structure, Fig. 13. The D–A interaction seems to have a crucial role in this case, given the EA nature of trisimide NG and the slight ED character of mesityl-functionalized HBC. As a result, the CT character of the heteroaggregates provokes a stabilization due to electrostatic dipole–dipole interactions between layers.

**Fig. 14:**
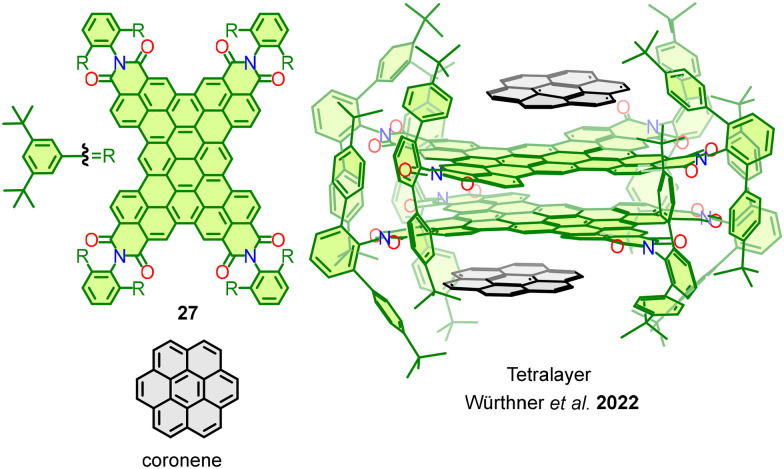
Tretralayer structure formed by two tetraimide nanographenes 27 and two coronene molecules.

In a subsequent study, authors synthesized a tetraimide nanographene 27 and evaluated its multilayer arrangement with different PAHs (Fig. 14).^80^ Several multilayered supramolecular complexes with coronene were identified by SCXRD. As mentioned, these complexes are stabilized by strong π–π and by intermolecular [CH⋯π] interactions, but also DFT calculations determined that electrostatic and dispersion forces play a major stabilizing role.

These publications indicate a significant through-space bilayer and multilayer interaction in nanographene derivatives with D–A character, which is ultimately responsible for the solid-state aggregation patterns.

**Fig. 15:**
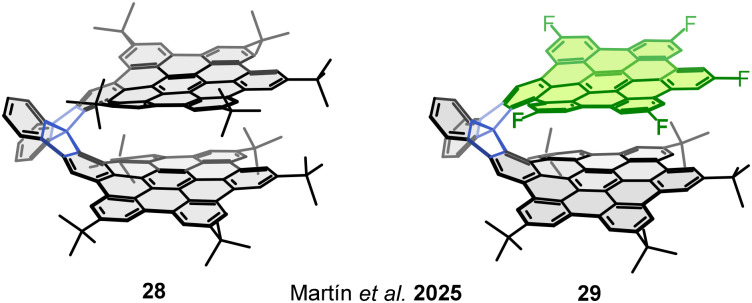
Spirobifluorene-linked molecular nanographenes 28 and fluorine-containing 29.

Considering the influence of the overlapping benzene rings in bilayer and multilayer nanographenes, our research group designed a bilayer molecular nanographene 28 in which the π-conjugation is minimized, if not supressed, and thus the through-bond electronic communication is residual (Fig. 15). The bilayer 3D structure of 28 is stabilized by strong π–π interactions occurring among 10 overlapping benzene rings – or two perylene subunits – and displays a 3.7 Å interlayer distance between the two graphene flakes (HBCs).^81^

This allows the interlayer communication and the expression of a bilayer effect like those observed in HBNGs 1, 3 and 4. The cyclic voltammetry analysis of 28 showed a first oxidation potential at 0.57 V *vs.* Fc/Fc^+^, which is significantly lower than that of benchmark *tert*-butyl-HBC (0.63 V). This behaviour can be exclusively attributed to the π–π interactions, given that other spirobifluorene-linked HBCs do not present a lowering in the first oxidation potential with respect to *tert*-butyl-HBC due to spiroconjugation.^82^ This finding supports the influence of bilayer interactions in the electronic properties of molecular nanographenes.

**Fig. 16:**
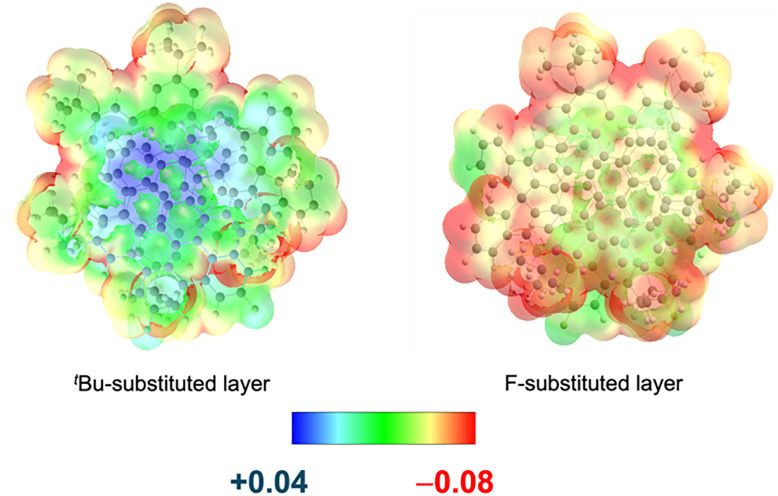
Calculated electrostatic potential surface of bilayer 29.

In an additional step, it seemed intriguing to generate systems of this kind incorporating both a defect and an excess of electron density and evaluate the through-space electronic communication. For this purpose, a D–A nanographene 29 bearing five electron-withdrawing fluorine atoms in one HBC and five electron-donating *tert*-butyl groups in the other HBC was synthesized (Fig. 15). The resulting molecule suffers from a ground-state electron transfer from the donor HBC to the acceptor HBC, resulting in a persistent zwitterionic open–shell nanographene species in which both a radical cation and a radical anion coexist. The electronic decoupling of both monoradicals was evidenced by EPR spectroscopy and theoretical calculations. The electrostatic potential surface (Fig. 16) reveals the distinct spatial distribution of charges across the layers: while the positive charge is localized at the center of the donor layer, the negative charge is mainly concentrated on the fluorine atoms of the acceptor layer. This different location of the charges explains the resistance to a charge recombination or back electron transfer process. Additional measurements confirmed the singular properties of this NG, which include high charge carrier mobility (Σ*μ* = 6 cm^2^ V^−1^ s^−1^) and an extremely narrow electrochemical HOMO–LUMO gap (0.63 eV). This constitutes an unprecedented behaviour in the field of molecular nanographenes, which directly arises from a bilayer disposition of two electronically modified graphene fragments.^77^

## Chemical reactivity of bilayer nanographenes

### Redox reactions in bilayer nanographenes

Given that the bilayer structure in nanographenes strongly influences their optoelectronic properties, redox reactions in these systems have also been explored.

**Fig. 17:**
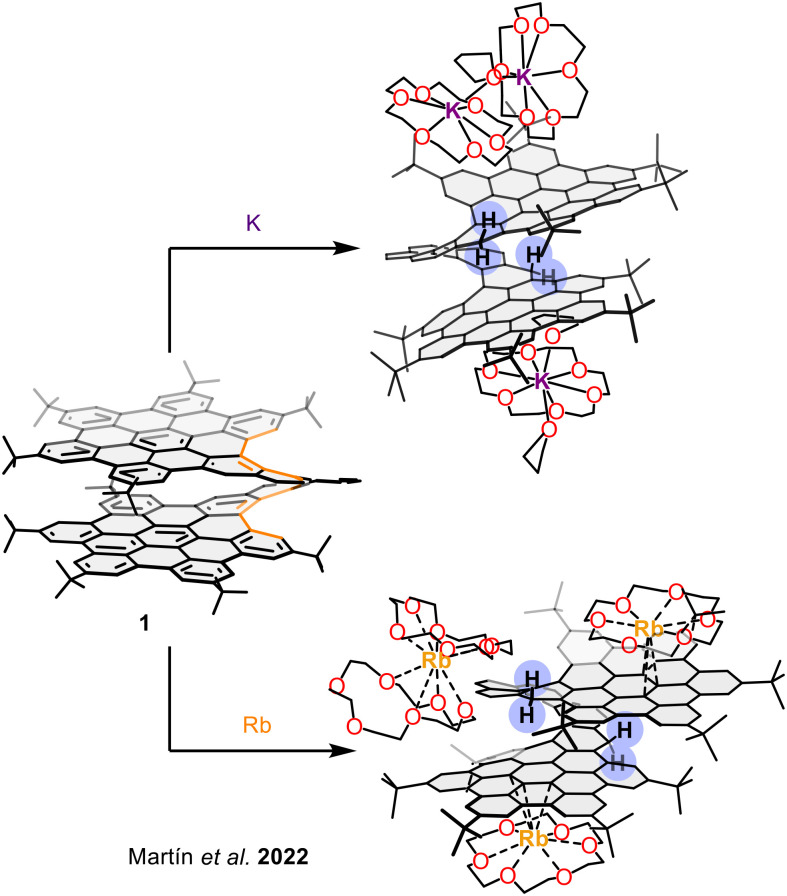
SCXRD structures of [1-H_2_]^3−^ after reduction with K and Rb metals.

A striking example of how bilayer architecture modulates redox behaviour is presented in the study by Fernández, Petrukhina, Martín and co-workers on helical bilayer nanographene 1. Upon chemical reduction with potassium and rubidium metals, a site-specific hydrogenation at the helicene edge was observed (Fig. 17), induced by electron localization in the helicene moiety of this bilayer NG.^83^ This process resulted in the formation of a rare radical trianion (1-H_2_^3−^), structurally characterized by X-ray diffraction. The bilayer configuration was critical in accommodating these redox-induced changes, as it enabled a pronounced slippage and compression of the layers due to increased electron density-the negative charge is spread over both HBCs-, while still maintaining the AA arrangement due to stabilizing π–π stacking.

**Fig. 18:**
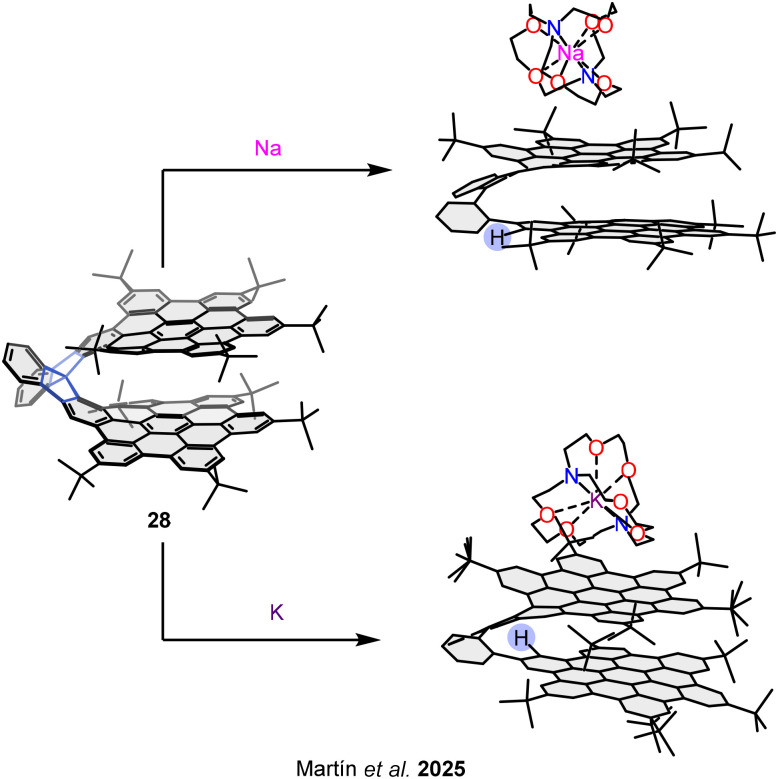
SCXRD structures of [28-H]^−^ after re duction with Na and K metals.

In a recent work, the reduction of bilayer spironanographene 28 was described.^84^ Upon one-electron reduction with sodium or potassium metals in the presence of [2.2.2]cryptand, the highly strained spirobifluorene core undergoes a C–C bond cleavage, accompanied by a site-specific hydrogenation to yield a monoanion ([28-H]^−^, Fig. 18). X-Ray crystallography confirmed the generation of a “naked” anion with broken spiro-linkage and enhanced aromaticity in the resulting indenyl-like five-membered ring. DFT calculations revealed that the strain within the bilayer direct the electron density localization to a specific carbon atom, triggering the selective hydrogenation. Notably, the bilayer's π–π interactions remain intact despite the structural rearrangement, contributing to the stability of the final anion.

These two studies showcase how bilayer-induced strain and π–π stacking geometry can direct localized electronic and structural responses to redox stimuli, offering new pathways for tuning the otherwise inaccessible transformations and electronic properties of molecular nanographenes.

### Bond formation between layers: radical reactivity

The formation of so-called pancake-bonded nanographenes, typically involving radical species, is emerging as a promising strategy for the construction of bilayer nanographene architectures. Although radical dimerization is a reversible process, the thermodynamic predominance of the bilayer form can be reinforced through favourable π–π interactions.

**Fig. 19:**
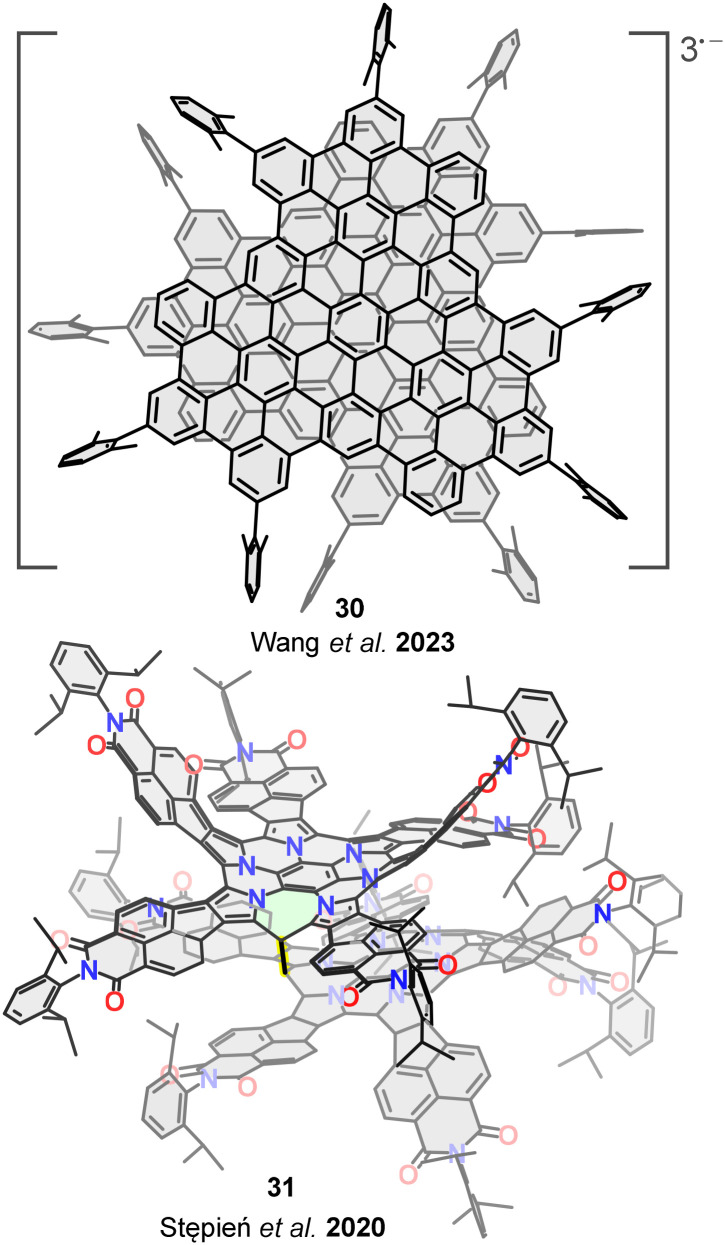
Open–shell bilayer nanographenes 30 and 31.

Following this strategy to access van der Waals bilayer nanographenes, Tan, Wang, and co-workers synthesized bilayer structure 30.^85^ Upon chemical reduction with potassium, this structure was converted into the triply charged radical anion 30^3−^ (Fig. 19, top). The resulting π-dimer radical anion is stabilized by an extensive 96-center, 3-electron pancake bond. Notably, the open–shell bilayer exhibits spin frustration, a property of significant interest in the field of magnetic materials. This suggests that upon chemical doping, π-extended nanographenes may exhibit features characteristic of quantum spin liquids.

In addition to π-dimerization, the more conventional σ-dimerization of open-shell molecules has also been harnessed to prepare bilayer systems. In this context, Stępień and co-workers reported bilayer structure 31,^86^ formed *via* a σ-bond between two radicaloid monomers based on a hexaazacoronene core functionalized with six annulated naphthalenemonoimide units (Fig. 19, bottom). The high thermodynamic stability of this dimeric bilayer arises from a combination of dispersion and covalent interactions, along with the steric protection afforded by bulky substituents. Remarkably, the dissociation of the bilayer into its open–shell monomers could be induced by UV or visible light irradiation, opening new possibilities for the design of magnetically responsive nanographenes.

**Fig. 20:**
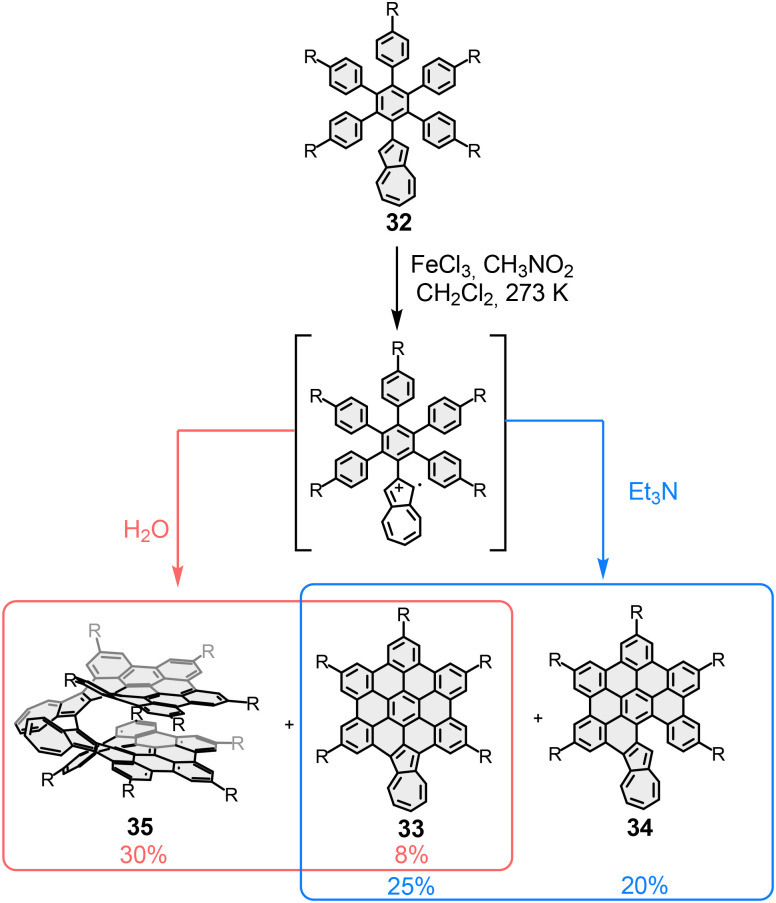
Scholl reaction of polyarene 32. Different quenching conditions conducted to the formation of monolayers 33 and 34, or the bilayer 35.

Very recently, Gong *et al.* reported another example of σ-bond formation between radicaloid nanographenes.^87^ In this case, the final Scholl reaction of polyarene 32 afforded either the monomers 33 and 34 (single-layer) or the bilayer dimer 35, depending on the quenching conditions of the Scholl reaction (Fig. 20). The dimerization process was promoted by the generation of the radical-cation of 34. A pronounced bilayer effect was observed in the electrochemical behavior: while monomer 34 exhibits a first oxidation potential at *E*^1/2^_ox1_ = 0.88 V, the corresponding value for the bilayer species 35 is *E*^1/2^_ox1_ = 0.57 V. Since through-bond electronic communication between the two fragments of the bilayer is interrupted, stabilization of the radical-cation formed upon oxidation arises solely from through-space electronic communication enabled by interlayer overlap.

### Bilayer nanographenes as molecular tweezers: complexation reaction with fullerenes

**Fig. 21:**
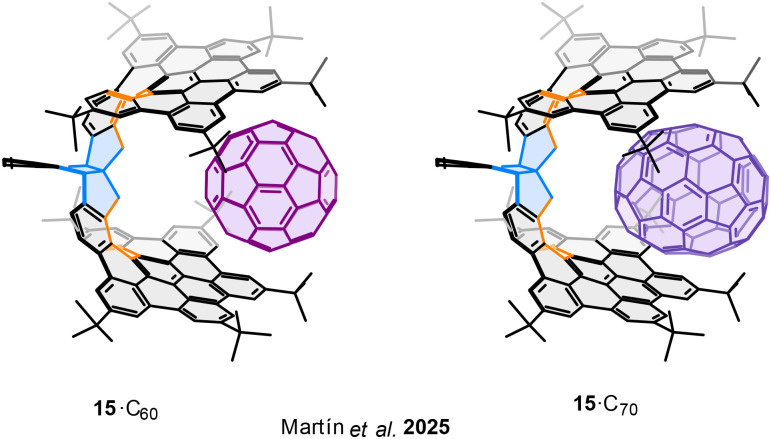
Supramolecular complexes of nanographene 15 with fullerenes C_60_ and C_70_.

A notable feature of bilayer nanographenes is their ability to act as molecular tweezers, enabling the formation of supramolecular complexes by encapsulating guest species between the two aromatic layers (Fig. 21). In this context, Martín and coworkers described the formation of host–guest complexes between the racemic bilayer nanographene 15 with fullerenes C_60_ and C_70_.^88^ Through NMR titration experiments, the authors determined the association constants for these complexes to be 61 M^−1^ for 15·C_60_ and 400 M^−1^ for 15·C_70_, respectively, indicating a marked selectivity toward the more π-extended C_70_ fullerene. This enhanced binding affinity is attributed to the better geometric and electronic complementarity between the bilayer cavity and the ellipsoidal shape of C_70_, which favours stronger π–π and CH–π van der Waals interactions.

Interestingly, the inherent chirality of 15 could play a significant role in the future for the selective recognition of chiral molecules, including enantiomerically pure fullerenes.

Other examples of fullerene complexation with carbon-based^89,90^ and nanographene-based^91–93^ macrocycles and cages have been reported in the literature; however, due to the significantly larger spatial separation between the HBC units in these systems, they do not behave as proper bilayer nanographenes. As such, they fall outside the scope of this review.

## Conclusions and perspectives

The recent advances in the synthesis and characterization of bilayer and multilayer nanographenes have unveiled the deep impact of π–π interlayer interactions on their optoelectronic and chiroptical properties. Among these, the so-called bilayer effect—arising from through-space π–π interactions between overlapping graphitized layers—emerges as a new and powerful structural parameter to tune the molecular behaviour. This effect is distinct from conventional approaches such as π-extension, heteroatom doping, or incorporation of non-hexagonal rings, offering a unique handle to modulate electronic communication and molecular functionality.

Our systematic investigation of helical bilayer nanographenes (HBNGs) clearly demonstrates that increasing the overlap between π-systems reduces the HOMO–LUMO gap, enhances charge carrier stabilization, and red-shifts the emission properties. Structures with high degrees of interlayer overlap, such as [9]HBNG, show mixed-valence behavior, strong electron donor character, and remarkably high circularly polarized luminescence (CPL) dissymmetry factors (over 10^−2^), underscoring the relevance of the bilayer configuration in controlling both ground- and excited-state properties. This effect extends beyond helical architectures, encompassing a wide variety of geometries, including spiro-linked, non-benzenoid, and donor–acceptor bilayer systems. In all these cases, the presence of well-defined face-to-face π–π stacking leads to enhanced optoelectronic responses, such as charge delocalization, suppressed non-radiative decay, and CPL activity. Moreover, the effect has been successfully translated to multilayer systems, where increased π-surface stacking correlates with progressive red shifts in emission and enhanced fluorescence quantum yields.

However, the preparation of enantiomerically pure HBNGs is still an underdeveloped area, which is critical for the application of these carbon nanostructures in novel technologies. In this regard, only very recently the first highly enantioselective synthesis of a HBNG was accomplished in our laboratory which, in turn, resulted to be synthetically demanding. Therefore, a simpler synthetic approach, based on the chemical resolution approach, involving a chiral auxiliary, has also been recently reported, where the chirality transfer for suitably functionalized binols to helicene led, in a straightforward manner, to chiral HBNGs with high ee values.

Finally, the singular topology and electronic effects stemming from HBNGs are responsible for their rich chemical reactivity. Thus, they exhibit interesting redox reactivity with alkaline metals leading to regioselective hydrogenation or C–C cleavage which strongly modify their chemical nature.

These findings collectively establish the bilayer effect as a fundamental design principle in nanographene chemistry. The overlap-driven modulation of redox potentials, electronic transitions, and chiroptical signals provides a robust framework for the development of functional nanocarbon materials with applications in organic electronics, chiral photonics, and quantum information.

Future efforts should aim to expand the scope of bilayer and multilayer nanographenes as programmable platforms for molecular functionality. Key synthetic challenges lie in two main areas: (i) increasing control over structural features such as π-conjugated extension, interlayer rotational degree and heteroatom incorporation (doping) within the graphene framework, and (ii) developing scalable stereoselective synthetic strategies to facilitate the production in view of future technological transfer. Overall, the precise engineering of multilayer molecular nanographenes will provide decisive insights on the structure-relationship properties both for fundamental science and for targeted advanced applications in fields such as electronics or biomedicine.

In summary, the deliberate construction of bilayer nanographenes, either *via* non-covalent or covalent strategies, offers not just structural elegance but also unprecedented control over molecular properties. The electronic communication between layers, thus, represents a further conceptual leap in the molecular design toolbox—introducing a vertical, through-space parameter capable of rivalling or even surpassing traditional two-dimensional modifications.

## Author contributions

All authors contributed to the conceptual development, critical analysis, and writing of the manuscript.

## Conflicts of interest

There are no conflicts to declare.

## Supplementary Material

CS-054-D4CS00804A-s001

## Data Availability

Data availability is not applicable to this article as no new data were created or analysed in this study. Supplementary information is available. See DOI: https://doi.org/10.1039/d4cs00804a.
