# Supplementary material for: Bilayer nanographenes: structure, properties, and synthetic challenges
Source: Chem Soc Rev. 2025 Oct 10;54(23):11089–104. doi: 10.1039/d4cs00804a (PMC12513161; doi:10.1039/d4cs00804a)
Supplement: CS-054-D4CS00804A-s001 [file CS-054-D4CS00804A-s001.pdf]

Supplementary Information (SI) for Chemical Society Reviews.
This journal is © The Royal Society of Chemistry 2025

Schematic Representation and Key Aspects of the Bilayer Effect in Nanographenes

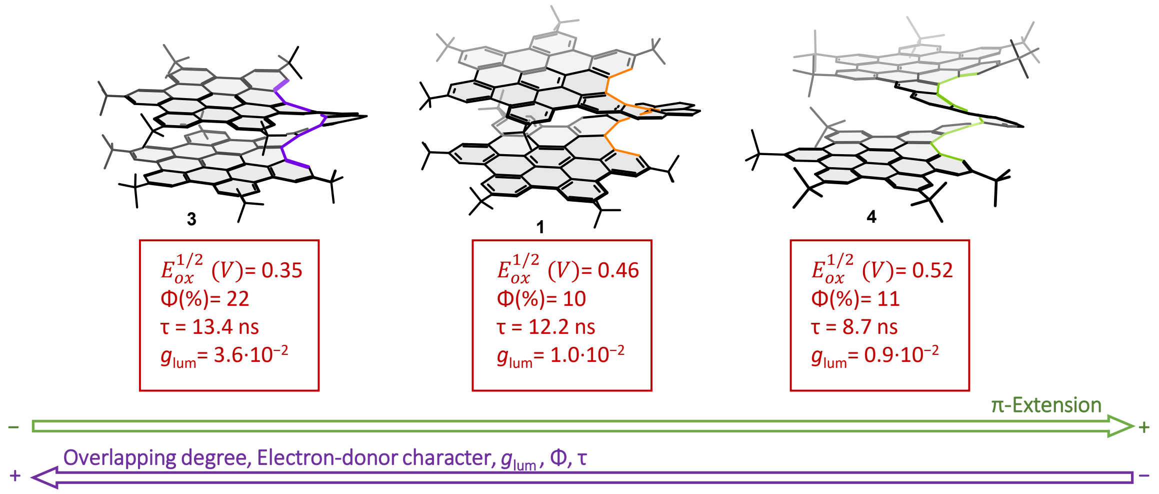

In these all-carbon structures (**1**, **3**, **4**) with minimal modifications, the primary structural feature variation lies in the overlapping degree between the layers. It is observed that increasing the number of rings involved in the overlapping increases the quantum yield (Φ) (which can be attributed to greater molecular rigidity and the consequent promotion of radiative deactivation pathways), and the photoluminescence lifetimes (τ). However, given the limited number of reported bilayer nanographenes in which minor structural modifications alter the overlapping degree in all-carbon compounds, the observed increase in quantum yield cannot yet be regarded as the dominant effect associated with enhanced bilayer character. The study of excited states remains inherently complex, as multiple factors must be taken into account. Moreover, all HBNGs generally display pronounced chiroptical properties, with dissymmetry factors (g_{abs}/g_{lum}) on the order of 10^{-3}/ 10^{-2}. Nevertheless, these properties depend on the relative orientation of the electric and magnetic transition dipole moments, and no direct correlation with bilayer character can currently be established.

Therefore, with this schematic explanation we want to highlight the most prominent effect resulting from the bilayer character, the enhanced electron donor character. To reveal structure/property relationship arising from the bilayer character, the first oxidation potentials are compared. Thus, the main "**bilayer effect**" −variation of the properties depending on the overlapping degree between layers in nanographenes− is reflected in the stabilization of the cation and radical-cation species between the layers. The larger the overlapping, the stronger the electron donor character (lower oxidation potentials). This trend is observed also in the electron acceptor character, and spectroelectrochemical measurements have been used for revealing mixed-valence band effects, however, not all manuscripts describe these properties.

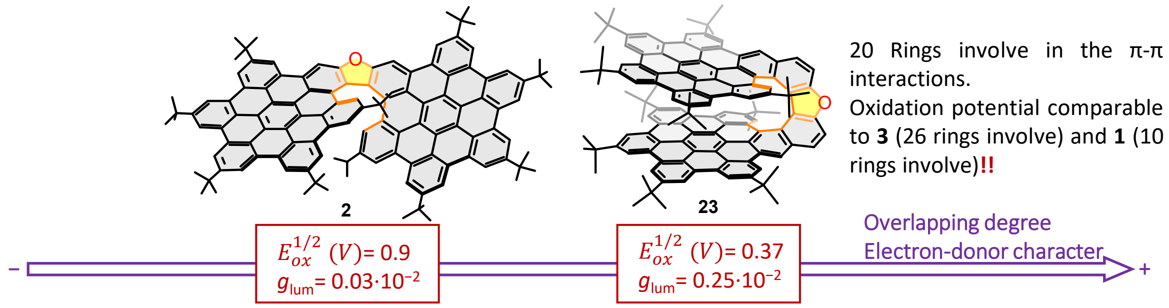

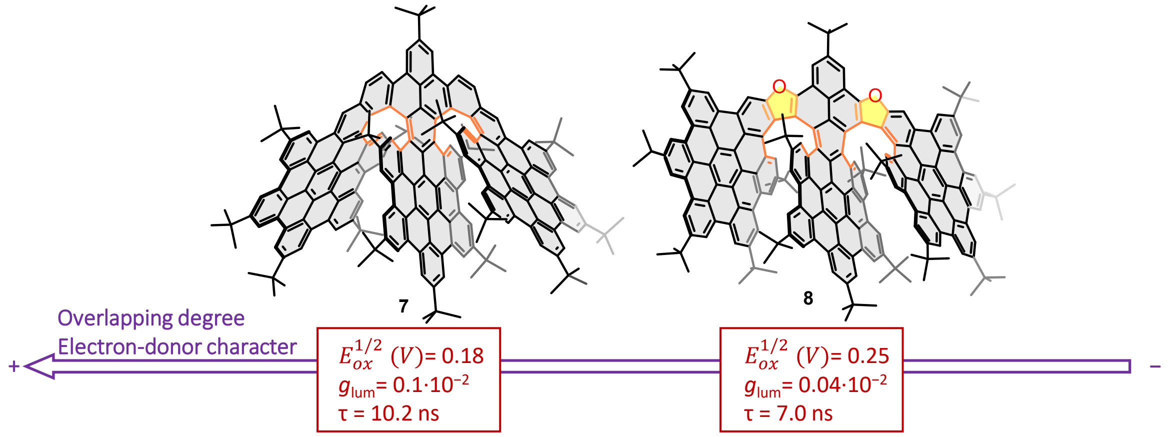

The five-membered rings embedded in the helicenes of trilayer graphene **8** open the inner rim of the helicene, thus decreasing the overlapping degree. The impact of this variation in the overlapping is clearly reflected in the electron donor character, the most overlapped structure (**7**) presents stronger electron donor character. For these structures the study of the photoluminescence also demonstrated the influence of the overlapping. The more overlapped structure **7** shows longer photoluminescence lifetime (10.2 ns) compared to **8** (7.0 ns).

Additionally, Table 1 summarizes the data for the Quantum yiels (Φ), luminescence dissymetry factors (g_{lum}) and first oxidation potentials ($E_{ox1}^{1/2}$ *vs* Fc/Fc$^+$) for the helical bilayer nanographenes discussed in the manuscript.

Table S1. ^{a}solvent $CHCl_3$, ^{b}solvent CH_2Cl_2, ^{c}solvent Toluene, ^{d}solvent Toluene/Acetonitrile 1:1.

Compound	Φ (%)	g_{lum}	$E_{ox1}^{1/2}$ (V)
^{t}Bu-HBC	2^a	–	0.75^d
**i2**	80^c	0.3·10^{-3}	0.9^b
**3**	22^a	3.6·10^{-2}	0.35^d
**1**	10^a	1·10^{-2}	0.46^d
**4**	11^a	9·10^{-3}	0.52^d
**5**	0,32^b	1.3·10^{-3}	0.2^b
**6**	10^b	4.5·10^{-2}	0.51^b
**7**	24^b	1·10^{-3}	0.18^b
**8**	37^b	4·10^{-4}	0.25^b
**9**	45^b	7.8·10^{-3}	0.45^b
**10**	74^b	2.6·10^{-3}	0.36^b
**11**	91^b	1.5·10^{-3}	0.27^b
**12**	41^b	8.7·10^{-3}	0.37^b
**13**	44^b	1.3·10^{-2}	0.36^b
**19**	31^a	4·10^{-2}	–
**20**	23^a	3.4·10^{-2}	–
**23**	30^a	2.5·10^{-3}	0.37^d
